# Peripheral blood lymphocyte to monocyte ratio recovery from low levels at diagnosis after completion of first line therapy predicts good clinical outcomes in patients with diffuse large B-cell lymphoma

**DOI:** 10.18632/oncotarget.14700

**Published:** 2017-01-17

**Authors:** Shujuan Zhou, Linglong Xu, Yongyong Ma, Liyuan Tang, Yu Zhang, Yifen Shi, Lan Sun, Yi Chen, Bin Liang, Yuhong Zhou, Kang Yu, Jianping Shen

**Affiliations:** ^1^ The First Clinical Medical College of Zhejiang Chinese Medical University, Hangzhou, Zhejiang 310006, P.R. China; ^2^ Department of Hematology, The First Affiliated Hospital of Wenzhou Medical University, Wenzhou, Zhejiang 325000, P.R. China; ^3^ Department of Hematology, The First Affiliated Hospital of Zhejiang Chinese Medical University, The First Clinical Medical College of Zhejiang Chinese Medical University, Hangzhou, Zhejiang 310006, P.R. China

**Keywords:** lymphocyte, monocyte, prognosis, diffuse large B-cell lymphoma

## Abstract

We retrospectively analyzed LMR at diagnosis and at completion of first-line therapy and prognosis in173 patients with DLBCL from 2005 to 2016. We found that patients with an LMR < 3.2 at diagnosis, as well as at completion of first-line therapy, had significantly lower PFS and OS rates than those with an LMR ≥ 3.2 (P<0.05). Patients with LMR that recovered from the low level at diagnosis showed superior overall survival (OS) (P=0.000) and progression-free survival (PFS) (P=0.001) compared with patients who failed to achieve a higher value at the completion of therapy. The multivariate analysis demonstrated that LMR values that did not increase upon completion of first-line therapy were an independent predictor for inferior OS (P=0.021) and PFS (P=0.046). In conclusion, LMR at diagnosis and at completion of first-line therapy is a simple biomarker to predict clinical outcomes in DLBCL. LMR recovery from low levels at diagnosis, irrespective of whether LMR reached the cutoff value, was associated with improved clinical outcomes.

## INTRODUCTION

Diffuse large B-cell lymphoma (DLBCL) is the most common form of lymphoma, accounting for 25% to 30% of all newly diagnosed cases of adult non-Hodgkin lymphoma (NHL). However, DLBCL is also classified as a heterogeneous entity, encompassing a number of morphologic variants, various biologic abnormalities, and variable clinical behaviors and responses to treatment [1]. A number of prognostic factors have been studied, such as the international prognostic index (IPI) [2], gene expression profiling (GEP) [3], immunohistochemistry-based detection of prognostic biomarkers [4, 5], and early interim analysis with positron emission tomography [6, 7] following the initiation of chemotherapy. Although these factors are useful for identifying patients who would benefit from standard therapy, many of these methods are costly [8], difficult to perform or not easily interpreted [9, 10], and they are tested at one point in time [11].

The peripheral blood lymphocyte/monocyte ratio (LMR) is a simple and effective biomarker for both host immune homeostasis (e.g., ALC, tumor-infiltrating lymphocytes) and the tumor microenvironment (e.g., AMC, tumor-associated macrophages). Previous studies have shown that the LMR obtained at diagnosis using the complete blood count (CBC) may predict clinical outcomes in DLBCL [12–15]; patients with a lower LMR (<3.8) showed a lower complete remission rate, 2-year PFS, and 3-year OS compared to patients with LMR ≥ 3.8 [16]. A limitation of the LMR is its inability to assess the host/tumor interaction after treatment, as it is performed at one point in time. However, the effects of LMR recovery from a low level upon diagnosis, after therapy, remain unclear. Thus, we explored whether peripheral LMR recovery from low levels at diagnosis, after completion of first-line therapy, can predict clinical outcomes in DLBCL.

## RESULTS

### Patient characteristics

There were 173 patients enrolled in this retrospective study. The median age for this cohort was 59 years (range, 18–80 years). The distribution of additional baseline characteristics of these patients are shown in Table 1. Median follow-up after diagnosis was 29 months for the entire cohort (range 6 to 120 months) and for censored observations. A total of 49 patients experienced relapse, disease progression, or death. The median PFS was 22 months (range 1–103 months), while the median OS was 29 months (range 6–120 months).

**Table 1:** Baseline patients’ characteristics based on LMR at diagnosis and at completion of therapy

Characteristics	Total (n=173)	LMR at diagnosis			LMR at completion of therapy		
		>3.2(n=79)	<3.2(n=94)	P-value	>3.2(n=73)	<3.2(n=100)	P-value
Male	95(55%)	40(51%)	55(59%)	0.300	43(59%)	52(52%)	0.440
Age (y), Median 59 (range, 18–80)				0.362			0.166
≥60	85(49%)	42(53%)	43(46%)		31(42%)	54(54%)	
<60	88(51%)	37(47%)	51(54%)		42(58%)	46(46%)	
Ann Arbor stage				0.009			0.390
I	39(23%)	23(29%)	16(17%)		16(22%)	23(23%)	
II	55(32%)	31(39%)	24(26%)		28(38%)	27(27%)	
III	19(11%)	6(8%)	13(14%)		8(11%)	11(11%)	
IV	60(34%)	19(24%)	41(43%)		21(29%)	39(39%)	
B symptoms				0.007			0.127
Presence	33(19%)	8(10%)	25(27%)		10(14%)	23(23%)	
Absence	140(81%)	71(90%)	69(73%)		63(86%)	77(77%)	
ECOG PS				0.005			0.129
0	55(32%)	32(41%)	23(24%)		30(41%)	25(25%)	
1	80(46%)	39(49%)	41(44%)		31(42%)	49(49%)	
2	24(14%)	5(6%)	19(20%)		8(11%)	16(16%)	
3 and 4	14(8%)	3(4%)	11(12%)		4(5%)	10(10%)	
Extranodal sites of disease				0.007			0.405
>1	41(24%)	11(%)	30(32%)		15(21%)	26(26%)	
≤1	132(76%)	68(%)	64(68%)		58(79%)	74(74%)	
IPI				0.000			0.130
0	34(20%)	19(24%)	15(16%)		16(22%)	18(18%)	
1	54(31%)	36(46%)	18(19%)		25(34%)	29(29%)	
2	36(21%)	12(15%)	24(26%)		19(26%)	17(%)	
3	30(17%)	10(13%)	20(21%)		9(12%)	21(21%)	
4 and 5	19(11%)	2(2%)	17(18%)		4(6%)	14(14%)	
WBC(×10^9^/L),median (range)	7.42 (2.43-27.50)	5.71±1.77	6.72±3.20	0.010	6.45±3.23	6.15±2.22	0.465
AMC(/μl), median (range)	0.59±0.40	0.40±0.14	0.75±0.46	0.000	0.65±0.53	0.63±0.43	0.786
ALC(/μl), median (range)	1.58±0.80	1.89±0.66	1.32±0.81	0.000	1.82±0.96	1.46±0.81	0.007
LDH >ULN *	64(37%)	16(20%)	48(51%)	0.02	23(32%)	41(41%)	0.264

^*^Serum LDH level >ULN (upper limit of normal), the normal range of LDH in our center is 0-250U/L.

Abbreviations: ECOG, Eastern Cooperative Oncology Group; PS, performance status; IPI, International Prognostic Index; LDH, lactate dehydrogenase; WBC, white blood cell; AMC, Absolute monocyte count ; ALC, Absolute lymphocyte count.

### LMR at diagnosis and upon completion of chemotherapy and clinical outcomes

**Figure 1:**
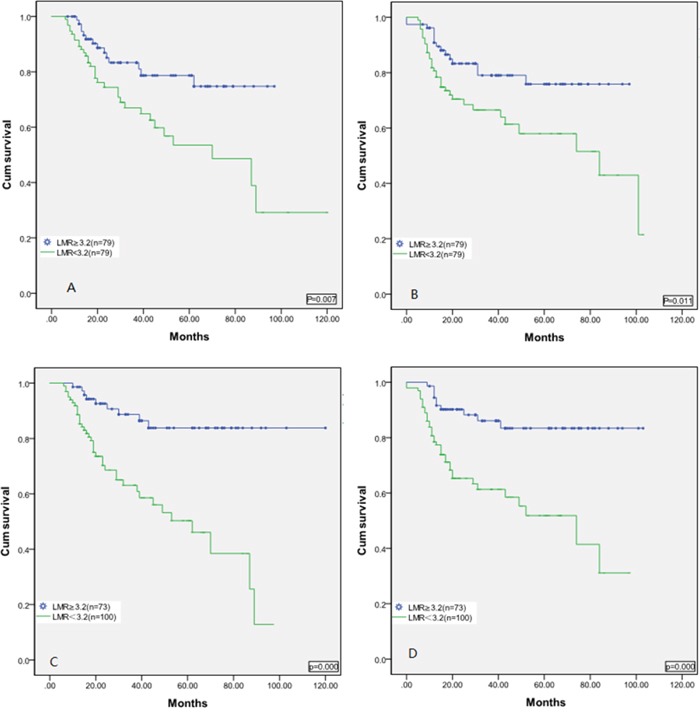
Kaplan-Meier estimates of overall survival A. and progression-free survival B. for the 173 DLBCL patients stratified by LMR at diagnosis Kaplan-Meier estimates of overall survival **C**. and progression-free survival **D**. for the 173 DLBCL patients stratified by LMR at the completion of therapy.

The median LMR at diagnosis was 3.06 (interquartile range, 0.17–11.55). A total of 79 (46%) patients had LMR ≥ 3.2 and 94 (54%) had LMR < 3.2 at diagnosis. LMR < 3.2 was significantly correlated with a higher Ann Arbor stage (P =0.009), IPI score (P=0.000), Eastern Cooperative Oncology Group Performance Status (ECOG PS) (P=0.005), and more extranodal sites of disease (P =0.007) (Table 1). As shown in Figure 1, patients with a low LMR < 3.2 had significantly lower OS rate [Figure 1(A), P=0.007] and PFS [Figure 1(B), P=0.011] than those with an LMR ≥ 3.2 at diagnosis.

The median LMR upon completion of therapy was 2.86 (interquartile range, 0.67–21.87). 81(46.8%) patients reached a higher LMR at the completion of therapy, but 93(53.4%) patients failed to do so. A total of 73 (42%) had LMR ≥ 3.2 and 100 patients (58%) had LMR < 3.2 upon completion of therapy. The patients’ baseline of LMR ≥ 3.2 and LMR < 3.2 upon completion of therapy was similar. However, as shown in Figure 1, patients with an LMR < 3.2 had significantly lower OS [Figure 1(C), P=0.000] and PFS [Figure 1(D), P=0.000] than those with an LMR ≥ 3.2 upon completion of therapy.

We found that patients who did not attain LMR ≥ 3.2 upon completion of therapy experienced inferior prognosis. Thus, we explored whether patients who started with a high LMR ≥ 3.2 at diagnosis but then obtained a low LMR < 3.2 upon completion of therapy showed inferior survival compared with patients who sustained a high LMR ≥ 3.2 upon completion of therapy. On the other hand, we explored whether patients who started with a low LMR < 3.2 at diagnosis but then gained a high LMR ≥ 3.2 showed superior survival compared to patients with a low LMR < 3.2 at the completion of therapy.

**Figure 2:**
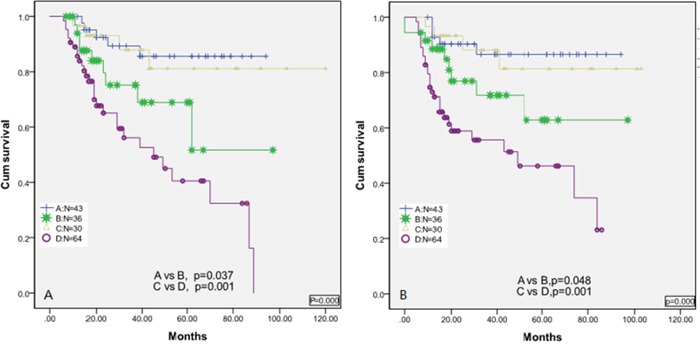
**A**. Overall survival based on group stratification; **B**. progression-free survival based on group stratification: Group A=patients with an LMR3.2 at diagnosis and at the completion of therapy; group B= patients with an LMR≥3.2 at diagnosis but then obtained an LMR < 3.2 at the completion of therapy; group C=patients with a LMR<3.2 at diagnosis but then gained an LMR≥3.2 at the completion of therapy; and group D =patients with a LMR <3.2 at diagnosis and at the completion of therapy.

To address these questions, patients were stratified into four groups. Group A included patients with an LMR ≥ 3.2 at diagnosis and at the completion of therapy; group B consisted of patients with an LMR ≥ 3.2 at diagnosis but then obtained an LMR < 3.2 at the completion of therapy; group C consisted of patients with a low LMR < 3.2 at diagnosis but then gained an LMR ≥ 3.2 at the completion of therapy; and group D consisted of patients with a low LMR < 3.2 at diagnosis and at the completion of therapy. As expected, based on cluster analysis, patients in group A experienced superior OS and PFS compared to the other groups [Figures 2(A) and 2(B)], and patients in group D experienced inferior OS and PFS compared to the other groups [Figures 2(A) and 2(B)]. However, group B experienced inferior OS and PFS compared to group A, suggesting that LMR < 3.2 upon completion of therapy despite an LMR ≥ 3.2 at diagnosis resulted in an inferior clinical outcome. In contrast, patients in group C experienced superior OS and PFS compared to group D, suggesting that an LMR ≥ 3.2 despite an LMR < 3.2 at diagnosis was associated with improved clinical outcomes.

Patients with LMR < 3.2 at diagnosis but LMR ≥ 3.2 upon completion of therapy showed superior OS and PFS. We next explored whether patients who started with a low LMR < 3.2 at diagnosis and then reached a higher value, but the value did not exceed 3.2 upon completion of therapy, showed superior survival compared to patients with a low LMR < 3.2 upon completion of therapy.

To address these questions, patients were stratified into three groups. Group I included patients with an LMR < 3.2 at diagnosis but then obtained an LMR ≥ 3.2 upon completion of therapy; group II consisted of patients with an LMR < 3.2 at diagnosis and who then reached a higher value, but the value failed to reach LMR ≥ 3.2 upon completion of therapy; group III consisted of patients with a low LMR < 3.2 upon diagnosis and who then failed to achieve a higher value upon completion of therapy.

**Figure 3:**
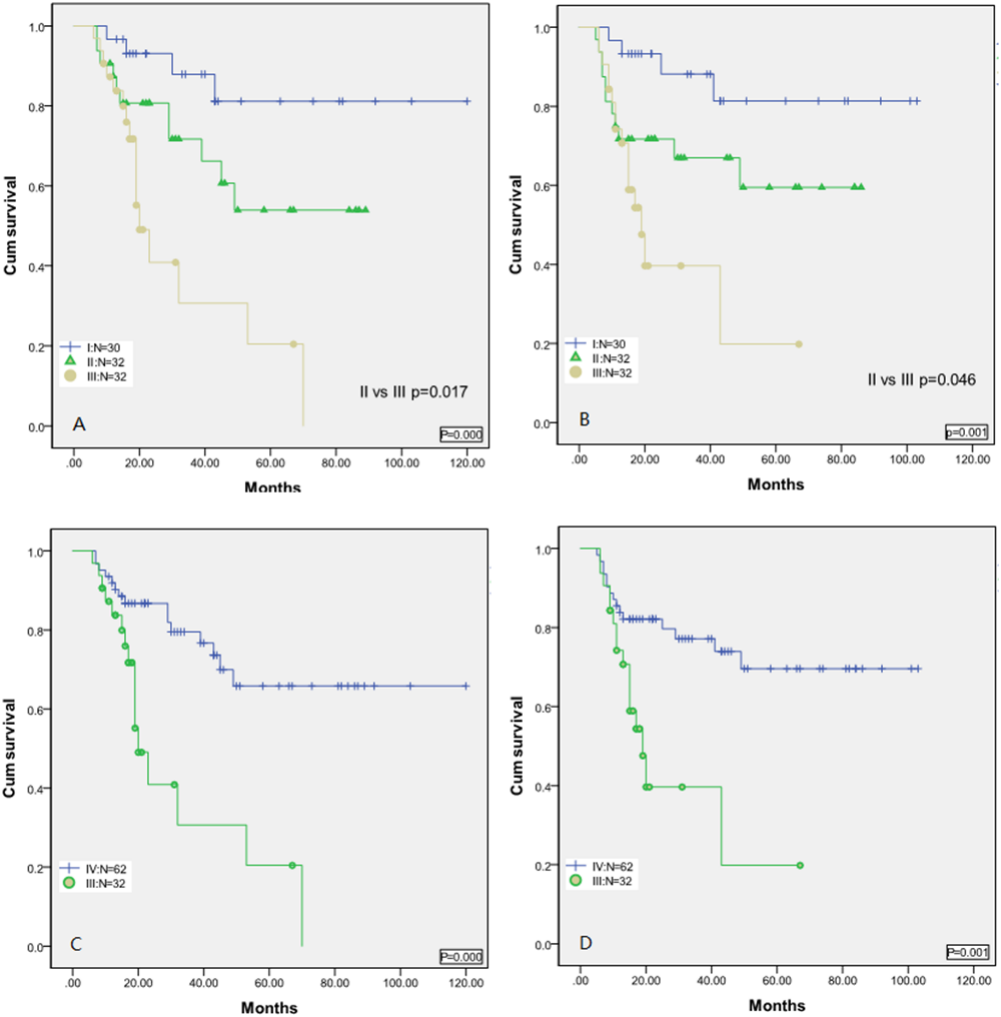
**A**. Overall survival based on group stratification; **B**. progression-free survival based on group stratification: Group I = patients with an LMR<3.2 at diagnosis but then obtained an LMR≥3.2 at the completion of therapy; group II =patients with an LMR<3.2 at diagnosis and then gained a higher LMR, but the LMR value failed to gain an LMR≥3.2 at the completion of therapy; group III =patients with a low LMR<3.2 at diagnosis but then failed to gain a higher value at the completion of therapy. **C**. Overall survival based on patients with LMR<3.2 at diagnosis but then obtained an higher LMR at the completion of therapy (group IV) versus group III. **D**. progression-free survival based on group IV versus group III.

As expected from the cluster analysis, patients in group I showed superior OS and PFS compared to the other groups [Figures 3(A) and 3(B)], and patients in group III experienced inferior OS and PFS compared to the other groups [Figures 3(A) and 3(B)]. However, group II experienced superior OS and PFS compared to group C, and had similar OS (P=0.105) and PFS (P=0.136), suggesting that attaining an LMR > baseline at diagnosis during treatment when LMR < 3.2 at diagnosis resulted in a superior clinical outcome.

### LMR increases versus LMR failed to increase upon completion of therapy when patients had a low LMR < 3.2 at diagnosis

Based on the cluster analysis showing that patients with a low LMR, who did not achieve a higher LMR value upon completion of therapy, experienced the most inferior clinical outcomes, patients were dichotomized into groups with LMR increases (group IV) versus LMR that did not increase (group II) upon completion of therapy. Both groups were balanced with regard to IPI risk factors, IPI score, and IPI score index (Table 2). Using Kaplan-Meier curves, patients with an increased LMR value experienced superior OS [Figure 3(C), P=0.000] and PFS [Figure 3(D), P=0.001] compared with patients with a low LMR, who failed to gain a higher value upon completion of therapy.

**Table 2:** Baseline patients’ characteristics with a low LMR<3.2 at diagnosis based on LMR elevated versus LMR failed to gain a higher value at the completion of therapy

Characteristic (n=94)	LMR elevate		P
	Yes (n=62)	No (n=32)	
Male	36(58%)	19(59%)	0.903
Age (y), Median 58 (range, 18–78)			0.190
≥60	25(40%)	18(56%)	
<60	37(60%)	14(44%)	
Ann Arbor stage			0.840
I	9(15%)	7(22%)	
II	16(26%)	8(25%)	
III	9(14%)	4(12%)	
IV	28(45%)	13(41%)	
B symptoms			0.368
Presence	19(31%)	7(22%)	
Absence	43(69%)	25(78%)	
ECOG PS			0.672
0	13(21%)	10(31%)	
1	28(45%)	13(41%)	
2	14(23%)	5(16%)	
3and4	7(11%)	4(12%)	
Extranodal sites of disease			0.571
>1	21(34%)	9(28%)	
≤1	41(66%)	23(72%)	
IPI			0.779
0	10(16%)	5(16%)	
1	10(16%)	8(25%)	
2	18(29%)	6(19%)	
3	13(21%)	7(21%)	
4 and 5	11(18%)	6(19%)	
WBC(×10^9^/L),median (range)	7.16±3.70	5.96±1.66	0.086
AMC(/μl), median (range)	0.72±0.54	0.62±0.21	0.096
ALC(/μl), median (range)	1.25±0.95	1.44±0.42	0.291
LDH >ULN *	34(55%)	14(44%)	0.385

^*^Serum LDH level >ULN (upper limit of normal), the normal range of LDH in our center is 0-250U/L.

Abbreviations: ECOG, Eastern Cooperative Oncology Group; PS, performance status; IPI, International Prognostic Index; LDH, lactate dehydrogenase; WBC, white blood cell;AMC, Absolute monocyte count; ALC, Absolute lymphocyte count.

### Univariate and multivariate analyses

The influence of the following variables on PFS and OS was evaluated in all 94 patients who had a low LMR<3.2 at diagnosis: LMR elevated or not, the LMR following the completion of therapy, the presence of B symptoms, and stage III/IV were significantly associated with clinical outcome upon univariate analysis (Table 3). Multivariate analysis revealed that only LMR elevated or not at the completion of first-line therapy remained an independent prognostic factor for OS and for PFS.

**Table 3:** Univariate analysis for overall survival and progression-free survival in patients with a low LMR<3.2 at diagnosis

Variable	Overall survival			Progression free survival		
	HR	95% CI	p -value	HR	95% CI	p -value
Female vs. male	1.554	0.756-3.194	0.230	1.396	0.676-2.885	0.367
Age<60 vs. ≥60 years	0.995	0.502-1.974	0.989	0.979	0.488-1.961	0.951
Stage I/II vs. III/IV	0.462	0.219-0.977	0.043	0.420	0.197-0.898	0.025
B symptoms presence vs.absence	0.409	0.204-0.818	0.012	0.457	0.228-0.914	0.027
ECOG PS≤1 vs. >1	0.698	0.336-1.452	0.336	0.604	0.289-1.26	0.179
Extranodal disease≤1 vs. >1	0.612	0.297-1.261	0.183	0.521	0.249-1.091	0.084
IPI≤1 vs.>1	0.637	0.30-1.352	0.240	0.568	0.264-1.221	0.147
LDH at diagnosis >ULN vs. normal	0.588	0.292-1.184	0.137	0.574	0.283-1.164	0.124
LDH following the completion of therapy >ULN vs. nomal	0.784	0.372-1.651	0.521	0.68	0.323-1.434	0.311
LMR at the completion of therapy ≥3.2vs. <3.2	0.205	0.072-0.585	0.003	0.191	0.066-0.550	0.002
LMR elevated vs. not	0.312	0.149-0.653	0.002	0.263	0.127-0.547	0.000

Abbreviations: ECOG, Eastern Cooperative Oncology Group; PS, performance status; IPI, International Prognostic Index; LDH, lactate dehydrogenase; ULN, upper limit of normal.

## DISCUSSION

DLBCL is classified as a heterogeneous entity; thus, numerous prognostic factors are required to guide physicians in the identification of patients who would benefit from standard therapy. However, all of these factors have limitations [17].

The LMR, a surrogate marker of both host immunity and the tumor microenvironment, is prognostic for survival of patients with DLBCL [15, 16, 18]. We evaluated the OS and PFS by comparing LMR upon completion of therapy to the baseline at diagnosis. We found that patients maintaining high LMR at diagnosis and at the completion of first-line chemotherapy constituted an independent prognostic factor for both PFS and OS in DLBCL patients. The low LMR group had a poorer prognosis. However, after the completion of first line chemotherapy, patients who achieved higher LMR compared to the level at diagnosis, irrespective of whether the LMR reached the cutoff value, showed improved prognosis. Conversely, patients with high LMR at diagnosis showed no association with the question of whether LMR increased after the completion of therapy, as long as LMR ≥ 3.2 after the completion of therapy (data not shown).

Based on previous studies, there are still several important questions to explore. For example, the cutoff values of the LMR at diagnosis, predicting clinical outcomes in patients with DLBCL, ranged from 2.1 to 3.8, as reported by Tadmor et al. [19] and Li et al. [16], respectively, although the authors used the same method [20]. We found that the different investigators used different cutoff values in calculating the LMR, so it is difficult to apply LMR to predict outcomes in practical clinical work. To account for this limitation, we compared the LMR upon completion of chemotherapy to the baseline, but not to the cutoff value, in patients with lower LMR at diagnosis. In addition, we did find it had prognostic significance. Host immunity (i.e., ALC) and tumor microenvironment (i.e., AMC) interaction during standard chemotherapy in DLBCL directly impacts survival.

It remains possible that the high LMR represents the normal immune status, and LMR ≥ 3.2 represents the normal LMR range. Thus, patients with high LMR at diagnosis have no correlation with whether LMR increased after the completion of therapy, as long as the LMR remained at high levels. It should be noted that LMR < 3.2 represents host immunological incompetence. Thus, after therapy, increased LMR is representative of immune recovery. Which also means the patients benefit from chemotherpy and they had a better prognosis. They only follow the NCCN guideline during the follow-up periods..

LMR failing to recover from the low level at diagnosis, after the completion of therapy, is associated with poor clinical outcomes. Thus, these patients may require more frequent evaluation, and salvage regimens are recommended in the case of relapse.

Our results are consistent with the study by Porrata et al. [21], who explored the LMR during each rituximab, cyclophosphamide, doxorubicin, vincristine, and prednisone (R-CHOP) cycle as a predictor for survival. They found that decreasing below an LMR ≥ 1.1 during treatment, despite having LMR ≥ 1.1 at diagnosis/cycle 1, resulted in an inferior clinical outcome, and patients reaching an LMR ≥ 1.1, despite having an LMR < 1.1 at diagnosis/cycle 1, was associated with improved clinical outcomes. A similar result can be attained from classical Hodgkin lymphoma during ABVD treatment cycles [22].

There are some limitations to our study. First, it was conducted in a single center, and it is a retrospective analysis on a small number of patients. Thus, further multicenter prospective studies containing more patients are required. Second, because some patients' detail pathology information were missing. So we did not divide the patients into two subtypes: germinal-center B-cell-like DLBCL (GCB-DLBCL) subtype and activated B-cell-like DLBCL (ABC-DLBCL) subtype. GCB subtype has better prognosis than ABC subtype. But the prognostic significance of LMR with respect to different cell-of-origin subtypes is inconsistent. In one study, L.F. Porrata [23] found that LMR was independent of cell of origin in DLBCL patients.. But in the other study, it was shown that low LMR is a negative prognostic marker for non-germinal center (GC) type DLBCL patients, but not in GC-type DLBCL patients [24].

In conclusion, LMR at diagnosis and following completion of first-line therapy is a simple and cost-effective biomarker predicting clinical outcomes and provides a platform for developing therapeutic interventions to manipulate the LMR and improve clinical outcomes in DLBCL patients. LMR recovery from the low level at diagnosis, irrespective of whether the LMR reached the cutoff value, was associated with improved clinical outcomes. However, further studies are required to confirm our results.

## MATERIALS AND METHODS

### Patients

The inclusion criteria were a diagnosis of *de novo* DLBCL, treatment with R-CHOP (rituximab, cyclophosphamide, hydroxydaunorubicin, vincristine, and prednisone), and follow-up at the First Affiliated Hospital of Wenzhou Medical University between January 2005 and June 2016. The dose of rituximab was 375 mg/m^2^ for all patients. Exclusion criteria were as follows: patients with primary central nervous system lymphoma, with transformed NHL, were treated with combined R-CHOP and irradiation, were positive for human immunodeficiency virus, or were lost to follow-up. From 2005 to 2016, 173 consecutive patients were enrolled and laboratory data of all patients were collected from electronic medical records. The research protocol was approved by the Ethics Committee of the First Affiliated Hospital of Wenzhou Medical University and performed in accordance with the principles of the Declaration of Helsinki, and written informed consent was obtained from every patient. They were followed until June 2016 to obtain survival information.

### Laboratory data

Lymphocyte and monocyte counts were obtained from standard complete blood cell count (CBC) data; each LMR was calculated by dividing the lymphocyte by the monocyte count. The LMR upon completion of first-line therapy was calculated when the CBC reached a plateau after the bone marrow had recovered from first-line therapy. It is the standard practice of our clinicians to obtain a CBC 3 months after completion of chemotherapy. Therefore, we used LMR data from the 3-month follow-up visits.

**Figure 4:**
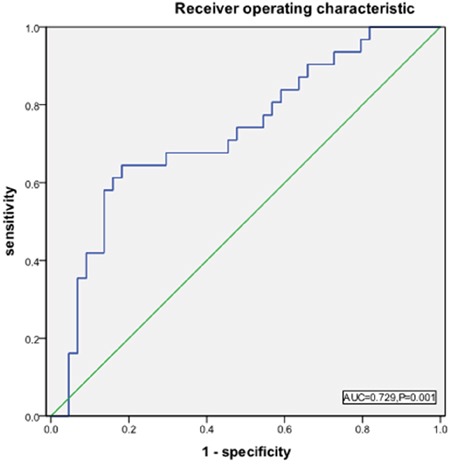
Receiver operating characteristic (ROC) curves analysis for LMR at diagnosis

The threshold of 3.2 was established as the maximum (sensitivity + specificity) point according to the area under curve (AUC) of the receiver operating characteristics curve (ROC; Figure 4). The binary clinical outcome (death/survival) was determined 5 years after diagnosis. Patients were categorized as “alive/censored” when the follow-up time was longer than 5 years and “dead” when they died before this time. Patients were further divided into two groups: low LMR group (LMR < 3.2) and high LMR group (LMR ≥ 3.2).

### Prognostic factors

The prognostic factors evaluated included the LMR (at diagnosis and at completion of first-line therapy); LDH level at diagnosis and at the completion of therapy; sex; IPI score; age of ≥ 60 years; more than one extranodal site of disease; an abnormal versus normal LDH level; ECOG PS > 1; and disease stage (I/II versus III/IV) at diagnosis.

### Statistical analysis

The definitions of the response and relapse criteria, OS and PFS, were those described in the guidelines of an NHL international workshop. OS and PFS were measured from the day following completion of first-line therapy. Statistical analysis was performed using IBM SPSS Statistic v21.0 (SPSS Inc., Chicago, IL, USA). Correlations of the LMR with clinical parameters were evaluated using the chi-square or Fisher's exact test. OS and PFS were analyzed using Kaplan–Meier curves, which were compared using the log-rank test. Multivariate prognostic analyses of OS and PFS were performed using Cox proportional hazards regression models. Categorical variables were compared using the chi-square test. P < 0.05 was considered statistically significant and all P values were two-tailed.

The authors thank the patients, their families, and all the investigators, including the physicians, nurses, and laboratory technicians in this study.

## References

[1] Habermann TM (2012). New developments in the management of diffuse large B-cell lymphoma. Hematology.

[2] (1993). A predictive model for aggressive non-Hodgkin's lymphoma. The International Non-Hodgkin's Lymphoma Prognostic Factors Project. N Engl J Med.

[3] Rosenwald A, Wright G, Chan WC, Connors JM, Campo E, Fisher RI, Gascoyne RD, Muller-Hermelink HK, Smeland EB, Giltnane JM, Hurt EM, Zhao H, Averett L (2002). The use of molecular profiling to predict survival after chemotherapy for diffuse large-B-cell lymphoma. N Engl J Med.

[4] Hans CP, Weisenburger DD, Greiner TC, Gascoyne RD, Delabie J, Ott G, Muller-Hermelink HK, Campo E, Braziel RM, Jaffe ES, Pan Z, Farinha P, Smith LM (2004). Confirmation of the molecular classification of diffuse large B-cell lymphoma by immunohistochemistry using a tissue microarray. Blood.

[5] Nyman H, Adde M, Karjalainen-Lindsberg ML, Taskinen M, Berglund M, Amini RM, Blomqvist C, Enblad G, Leppa S (2007). Prognostic impact of immunohistochemically defined germinal center phenotype in diffuse large B-cell lymphoma patients treated with immunochemotherapy. Blood.

[6] Mikhaeel NG (2009). Interim fluorodeoxyglucose positron emission tomography for early response assessment in diffuse large B cell lymphoma: where are we now?. Leuk Lymphoma.

[7] Duhrsen U, Huttmann A, Jockel KH, Muller S (2009). Positron emission tomography guided therapy of aggressive non-Hodgkin lymphomas--the PETAL trial. Leuk Lymphoma.

[8] Cerci JJ, Trindade E, Pracchia LF, Pitella FA, Linardi CC, Soares J, Delbeke D, Topfer LA, Buccheri V, Meneghetti JC (2010). Cost effectiveness of positron emission tomography in patients with Hodgkin's lymphoma in unconfirmed complete remission or partial remission after first-line therapy. J Clin Oncol.

[9] Choi WW, Weisenburger DD, Greiner TC, Piris MA, Banham AH, Delabie J, Braziel RM, Geng H, Iqbal J, Lenz G, Vose JM, Hans CP, Fu K (2009). A new immunostain algorithm classifies diffuse large B-cell lymphoma into molecular subtypes with high accuracy. Clin Cancer Res.

[10] de Jong D, Rosenwald A, Chhanabhai M, Gaulard P, Klapper W, Lee A, Sander B, Thorns C, Campo E, Molina T, Norton A, Hagenbeek A, Horning S (2007). Immunohistochemical prognostic markers in diffuse large B-cell lymphoma: validation of tissue microarray as a prerequisite for broad clinical applications--a study from the Lunenburg Lymphoma Biomarker Consortium. J Clin Oncol.

[11] Porrata LF, Rsitow K, Inwards DJ, Ansell SM, Micallef IN, Johnston PB, Habermann TM, Witzig TE, Colgan JP, Nowakowski GS, Thompson CA, Markovic SN (2010). Lymphopenia assessed during routine follow-up after immunochemotherapy (R-CHOP) is a risk factor for predicting relapse in patients with diffuse large B-cell lymphoma. Leukemia.

[12] Belotti A, Doni E, Bolis S, Rossini F, Casaroli I, Pezzatti S, Pogliani EM, Pioltelli PE (2015). Peripheral blood lymphocyte/monocyte ratio predicts outcome in follicular lymphoma and in diffuse large B-cell lymphoma patients in the rituximab era. Clin Lymphoma Myeloma Leuk.

[13] Markovic O, Popovic L, Marisavljevic D, Jovanovic D, Filipovic B, Stanisavljevic D, Matovina-Brko G, Hajder J, Matkovic T, Zivkovic R, Stanisavljevic N, Todorovic M, Petrovic D (2014). Comparison of prognostic impact of absolute lymphocyte count, absolute monocyte count, absolute lymphocyte count/absolute monocyte count prognostic score and ratio in patients with diffuse large B cell lymphoma. Eur J Intern Med.

[14] Li ZM, Huang JJ, Xia Y, Sun J, Huang Y, Wang Y, Zhu YJ, Li YJ, Zhao W, Wei WX, Lin TY, Huang HQ, Jiang WQ (2012). Blood lymphocyte-to-monocyte ratio identifies high-risk patients in diffuse large B-cell lymphoma treated with R-CHOP. PLoS One.

[15] Rambaldi A, Boschini C, Gritti G, Delaini F, Oldani E, Rossi A, Barbui AM, Caracciolo D, Ladetto M, Gueli A, De Crescenzo A, Passera R, Devizzi L (2013). The lymphocyte to monocyte ratio improves the IPI-risk definition of diffuse large B-cell lymphoma when rituximab is added to chemotherapy. Am J Hematol.

[16] Li YL, Pan YY, Jiao Y, Ning J, Fan YG, Zhai ZM (2014). Peripheral blood lymphocyte/monocyte ratio predicts outcome for patients with diffuse large B cell lymphoma after standard first-line regimens. Ann Hematol.

[17] Cheson BD, Fisher RI, Barrington SF, Cavalli F, Schwartz LH, Zucca E, Lister TA, Alliance, Australasian Leukaemia and Lymphoma Group; Eastern Cooperative Oncology Group; European Mantle Cell Lymphoma Consortium; Italian Lymphoma Foundation; European Organisation for Research; Treatment of Cancer/Dutch Hemato-Oncology Group (2014). Recommendations for initial evaluation, staging, and response assessment of Hodgkin and non-Hodgkin lymphoma: the Lugano classification. J Clin Oncol.

[18] Li YL, Gu KS, Pan YY, Jiao Y, Zhai ZM (2014). Peripheral blood lymphocyte/monocyte ratio at the time of first relapse predicts outcome for patients with relapsed or primary refractory diffuse large B-cell lymphoma. BMC Cancer.

[19] Tadmor T, Bari A, Sacchi S, Marcheselli L, Liardo EV, Avivi I, Benyamini N, Attias D, Pozzi S, Cox MC, Baldini L, Brugiatelli M, Federico M (2014). Monocyte count at diagnosis is a prognostic parameter in diffuse large B-cell lymphoma: results from a large multicenter study involving 1191 patients in the pre- and post-rituximab era. Haematologica.

[20] Tzankov A, Zlobec I, Went P, Robl H, Hoeller S, Dirnhofer S (2010). Prognostic immunophenotypic biomarker studies in diffuse large B cell lymphoma with special emphasis on rational determination of cut-off scores. Leuk Lymphoma.

[21] Porrata LF, Ristow KM, Habermann TM, Witzig TE, Colgan JP, Inwards DJ, Ansell SM, Micallef IN, Johnston PB, Nowakowski G, Thompson CA, Markovic SN (2014). Peripheral blood absolute lymphocyte/monocyte ratio during rituximab, cyclophosphamide, doxorubicin, vincristine and prednisone treatment cycles predicts clinical outcomes in diffuse large B-cell lymphoma. Leuk Lymphoma.

[22] Porrata LF, Ristow KM, Habermann TM, Macon WR, Witzig TE, Colgan JP, Inwards DJ, Ansell SM, Micallef IN, Johnston PB, Nowakowski G, Thompson CA, Markovic SN (2013). Peripheral blood absolute lymphocyte/monocyte ratio recovery during ABVD treatment cycles predicts clinical outcomes in classical Hodgkin lymphoma. Blood Cancer J.

[23] Porrata LF, Ristow K, Habermann TM, Ozsan N, Dogan A, Macon W, Colgan JP, Witzig TE, Inwards DJ, Ansell SM, Micallef IN, Johnston PB, Nowakowski GS (2012). Absolute monocyte/lymphocyte count prognostic score is independent of immunohistochemically determined cell of origin in predicting survival in diffuse large B-cell lymphoma. Leuk Lymphoma.

[24] Wei X, Huang F, Wei Y, Jing H, Xie M, Hao X, Feng R (2014). Low lymphocyte-to-monocyte ratio predicts unfavorable prognosis in non-germinal center type diffuse large B-cell lymphoma. Leuk Res.

